# A haplodiploid mite adjusts fecundity and sex ratio in response to density changes during the reproductive period

**DOI:** 10.1007/s10493-022-00749-0

**Published:** 2022-10-15

**Authors:** Nuwan Weerawansha, Qiao Wang, Xiong Zhao He

**Affiliations:** 1 School of Agriculture and Environment, Massey University, Private Bag 11222, Palmerston North, New Zealand; 2 Faculty of Animal Science and Export Agriculture, Uva Wellassa University of Sri Lanka, Passara Road, Badulla, 90000 Sri Lanka

**Keywords:** Spider mite, Fecundity, Sex ratio, Resource competition, Social environment

## Abstract

Population density is one of the main socio-environmental factors that have critical impacts on reproduction of animals. Consequently, they need to adjust their reproductive strategies in response to changes of local population density. In this study we used a haplodiploid spider mite, *Tetranychus ludeni* Zacher (Acari: Tetranychidae), to test how population density dynamics during the reproductive period altered female reproductive performance. We demonstrate that females produced fewer eggs with a significantly higher female-biased sex ratio in dense populations. Reducing fecundity and increasing daughter production in a dense environment could be an advantageous strategy to minimise the intensity of local food competition. However, females also reduced their fecundity after arrival in a new site of larger area from a dense population, which may be associated with higher web production costs because females need to produce more webs to cover the larger area. There was no trade-off between egg number and size, and egg size had little impact on reproductive fitness. Therefore, *T. ludeni* females could adapt to the shift of population density during their reproductive period by manipulating the fecundity and offspring sex ratio but not the egg size.

## Introduction

Population density is one of the major components of social environments that can affect population dynamics. A local population density may vary over time due to aggregation (immigration), dispersal (emigration), or mortality (Roeder 1992; Roff 1992; Stearns 1992; Bowman et al. 2002; Schausberger et al. 2021). Animals may aggregate in a habitat to increase reproduction opportunities (e.g., Snead and Alcock 1985; Bengtsson 2008; Wheeler and Jr Welsh 2008; Le Goff et al. 2010; Pérez-González et al. 2010; Bonsignore and Jones 2014; DeVries et al. 2017. Dar et al. 2021) or to reduce predation risk (e.g., Spieler 2003; Morrell and James 2008; Yano 2012; Clotuche et al. 2014). However, aggregation may raise population density in the habitat, leading to intensive resource competition and reducing reproductive fitness (Li and Zhang 2021; Weerawansha et al. 2020, 2022). In this scenario, the reproductive females would disperse to seek new habitats for the next generation (Schaub and von Hirschheydt 2009; Azandémè-Hounmalon et al. 2014; Lutz et al. 2015; Kingma et al. 2017; Kusch et al. 2020; Manguette et al. 2020; Vaishali and Krushnamegh 2020; Schausberger et al. 2021; Zhou et al. 2021). With a few exceptions (e.g., Roeder 1992; Fox et al. 1997; Matsuura and Kobayashi 2010; Maenoa et al. 2020), studies on the effects of population density on reproductive plasticity have been carried out under constant population densities during female breeding time (e.g., Wrensch and Young 1978; Fischer et al. 2011; Weerawansha et al. 2020, 2022). To date, it is still unclear how females alter their reproductive strategies in response to the varying population density during their reproductive life.

After settling in new habitats, females are expected to adjust their reproductive strategies to optimize their fitness (Roff 1992; Stearns 1992; West et al. 2005; Fischer et al. 2011; Bowers et al. 2017; Maenoa et al. 2020; Weerawansha et al. 2022). For example, if the population is dense, females may lay fewer (van Noordwijk and de Jong 1986; Khan et al. 2018; Li and Zhang 2021) but larger eggs (Parker and Begon 1986; Sibly et al. 1988; Fischer et al. 2011), trading-offs the number with size of eggs to make best utilization of limited resources and maximise offspring fitness (Smith and Fretwell 1974; Parker and Begon 1986; Stearns 1992; Fox and Czesak 2000; Fischer et al. 2011; Macke et al. 2012; Walzer and Schausberger 2015; Maenoa et al. 2020). In species with sexual size dimorphism, resource-deficient females either reduce the egg size of the larger sex (Fox and Czesak 2000; Walzer and Schausberger 2013, 2015) or produce fewer eggs of the larger sex (Trivers and Willard 1973; Charnov 1982; Walzer and Schausberger 2015) to optimize their reproductive fitness returns. Moreover, if one sex is dispersive and the other is philopatric, females often skew investment towards philopatric offspring when local resources are abundant but allocate more resources to the dispersive sex when local resources are deficient (Clark 1978; Silk 1983, 1984; West et al. 2005; Hjernquist et al. 2009; West 2009).

Spider mites (Acari: Tetranychidae) are phytophagous invertebrates, often living as groups (Helle and Sabelis 1985; Le Goff et al. 2010; Schausberger et al. 2021) in discrete patches (Mitchell 1973; Nachappa et al. 2011; Sarwar 2013). Female adults are larger than male adults (Mitchell 1973) and thus more likely to compete for food with their mothers or siblings (Young et al. 1986). However, female adults, rather than male adults and immature nymphs, may disperse to found new colonies (Mitchell 1973; Brandenburg and Kennedy 1982) especially when the populations are crowded or when food is insufficient or poor in quality (Suski and Naegele 1968; McEnroe 1969). As spider mites are haplodiploid, mated females can manipulate offspring sex ratio by fertilizing relatively larger eggs that develop to daughters (Young et al. 1986; Roeder et al. 1996; Macke et al. 2011). It has been reported that females produce fewer eggs with more dispersing daughters in large and dense populations to reduce local competition for food (Weerawansha et al. 2022). Moreover, spider mites aggregate and cooperate in spinning silk webs for dispersal and protection against environmental hazards (Le Goff et al. 2010; Yano 2012), and group-living females produce more silk and lay more eggs per mite than single females (Le Goff et al. 2010). Therefore, spider mites should be able to adjust offspring sex ratio in response to the social environments.

Here, we used an invasive pest spider mite, *Tetranychus ludeni* Zacher (Zhang 2003), to examine how changes in population density during female reproductive life altered egg production and sex allocation. We simulated the aggregation by moving females from low to high population density and the dispersal by shifting females from high to low population density. We recorded the number and size of eggs laid and offspring sex ratio (i.e., proportion of daughters) before and after density changes. Based on the knowledge outlined above, we hypothesize that (1) females lay fewer but larger eggs and produce offspring with a more female-biased sex ratio in response to the aggregation scenario, and (2) the opposite case occurs in response to the dispersal scenario. This study provides insight into the mechanisms behind the adjustment of fecundity and sex ratio in response to the varying social environments.

## Materials and methods

### Mite colony

We maintained a colony of *T. ludeni* on kidney bean plants (*Phaseolus vulgaris* L.) in the laboratory—and carried out the experiment—at 25 ± 1 ºC, 40 ± 10% RH and L16:D8 h photoperiod. We used the first expanded leaves of 1- to 2-week-old plants for the experiment.

### Experiment

**Fig. 1:**
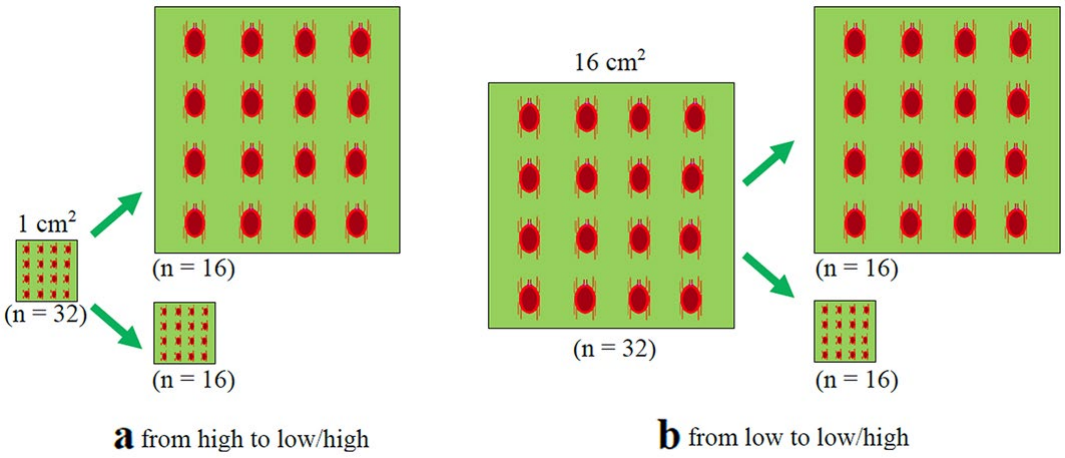
Diagram of experimental design to test the effect of population density shift on reproduction in *Tetranychus ludeni*: **a** from high to low/high density (Treatment 1) and **b** from low to low/high density (Treatment 2). *n* number of leaf discs (replicates)

To determine how females adjusted their fecundity and sex allocation in response to population density dynamics in *T. ludeni*, we set up two treatments, each with 32 leaf squares as replicates. Treatments 1 and 2 tested the effects of density changes from high to low (Fig. 1a) and from low to high (Fig. 1b), respectively. Briefly, we randomly selected the quiescent female deutonymphs just before emergence (silvery in colour) from the colony. We individually transferred them onto 1-cm^2^ leaf squares placed upside down on a water-saturated cotton pad in a Petri dish (9.5 cm diameter, 1 cm high) with a hole (1 cm diameter) in the middle of the lid covered by a fine metal mesh (aperture size 0.25 × 0.25 mm). We then introduced a newly emerged virgin male adult produced by virgin females onto each square. We monitored the pair until the end of copulation, after which time, we removed the male. For each replicate in Treatment 1, we introduced 16 newly mated females onto a 1-cm^2^ clean leaf square and allowed them to stay on the square for 1 day. We then transferred them to a new square daily for two consecutive days. On the 4th day, we randomly selected 16 of the 32 leaf squares and transferred mites from each leaf square onto a new 16-cm^2^ leaf square (from high to low density) and those from each of the remaining 16 leaf squares onto a new 1-cm^2^ leaf square (from hight to high density as control) daily for three consecutive days. The same procedure was carried out for Treatment 2 except that we transferred mites from low to high density and from low to low density (as control).

We checked each leaf square twice a day during the six oviposition days and replaced any dead females immediately with females of the same age and social experience. We recorded the number of eggs laid on each leaf square. To determine the egg size, we randomly selected 30 eggs from each leaf square and individually measured their diameter under a stereomicroscope (Leica MZ12, Germany) connected to a digital camera (Olympus SC30, Japan) and imaging software (CellSens GS-ST-v.1.7, Olympus, Japan). We calculated the egg radius (*r* = diameter/2) and egg size (volume = 4/3*πr*^3^). After eggs hatched, we transferred all live individuals onto a clean leaf square of the same size once every 5 days and recorded the sex of newly emerged adults.

### Statistical analysis

We analysed all data using SAS v.9.4 with a rejection threshold set at α = 0.05. Data on the number of eggs laid and egg size were normally distributed (Shapiro-Wilk test; UNIVARIATE procedure). We analysed the data on egg number and size using a linear mixed model (GLM procedure) with treatment (i.e., density shift) as a main factor and replicate as a random factor, and a Tukey-Kramer test for multiple comparisons. The mean egg size and number for each female before and after density shift were calculated and used for analysis. The data on sex ratio (proportion of daughters) were analysed by a generalized linear model (GLIMMIX procedure) with a binomial distribution and a *Logit* link function after the model, and a Tukey-Kramer test was applied for multiple comparisons. A general linear regression model (GLM procedure) was applied to determine the relationships between egg size and number, between immature survival rate and egg size, and between sex ratio and egg size. The mean egg size and number, immature survival rate, and sex ratio for each female were used for regressions.

## Results

### Effect of population density shifts on fecundity, egg size and immature survival

**Fig. 2:**
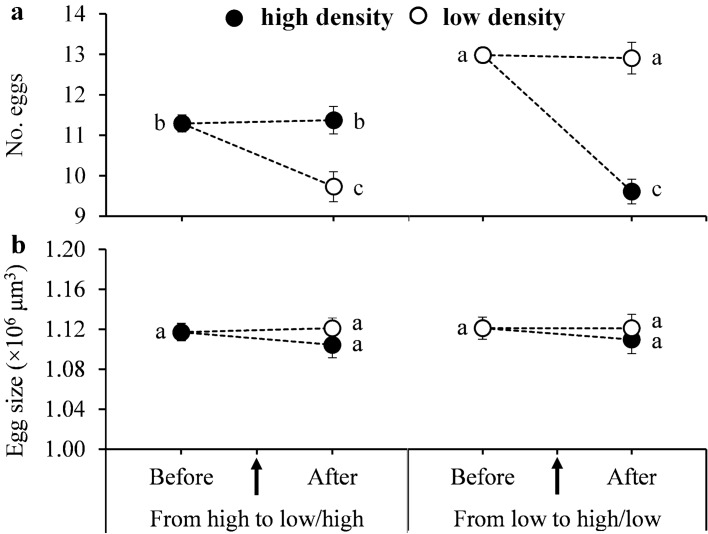
Effects of female population shift between high (black dots) and low (white dots) densities on the mean (± SE) number of eggs laid (**a**) and egg size (**b**) in *Tetranychus ludeni*. Means within a panel with the same letters are not significantly different (Tukey-Kramer test: *P* > 0.05). Female population density shift occurred on the 4th day of oviposition period

**Fig. 3:**
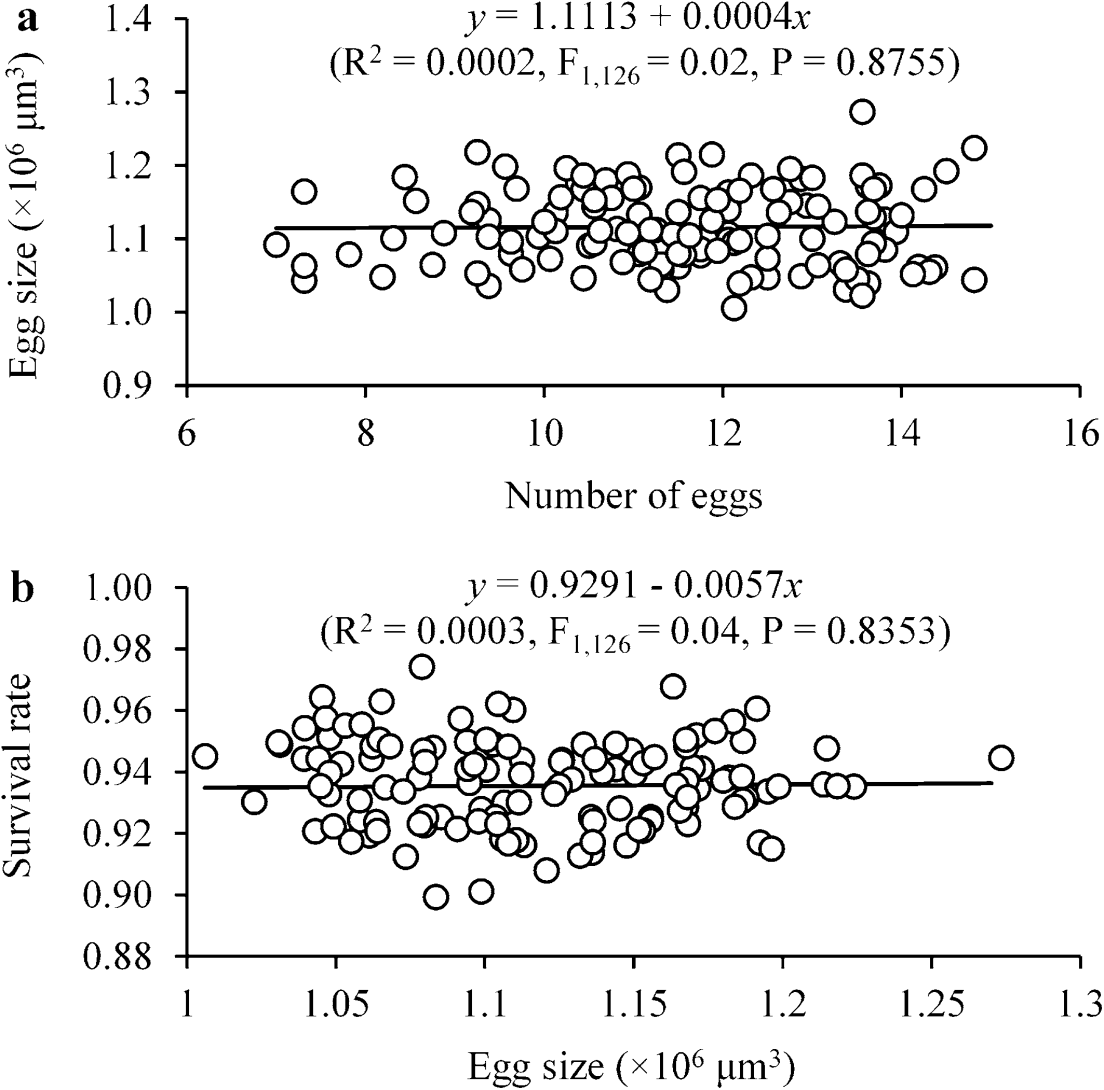
Relationships between egg size and number of eggs laid (**a**) and between egg size and immature survival rate (**b**) in *Tetranychus ludeni*

Our results show that females at low density laid significantly more eggs than at high density before density shift but laid significantly fewer eggs after the shift regardless of whether it was from high to low or from low to high (*F*_5,107_ = 28.79, *P* < 0.0001) (Fig. 2a). If the shift occurred at the same density levels, females produced similar numbers of eggs before and after shift (Fig. 2a). However, population density and its shift had no significant impact on egg size (*F*_5,107_ = 0.37, *P* = 0.87) (Fig. 2b). Moreover, increasing number of eggs laid did not significantly reduce the egg size (Fig. 3a) and egg size had no significant impact on immature survival rate (Fig. 3b).

### Effect of population density shifts on sex allocation

**Fig. 4:**
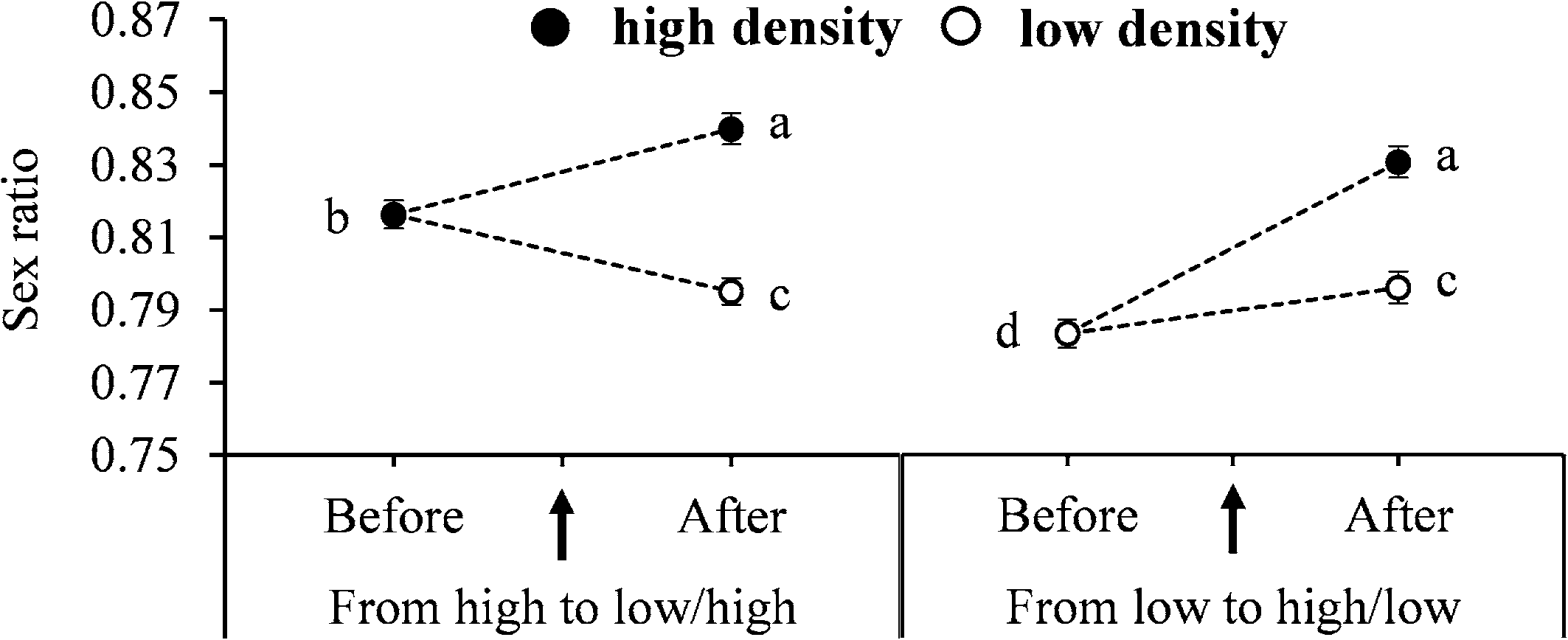
Effects of female population shift between high (black dots) and low (white dots) densities on the mean (± SE) sex ratio (proportion of females) in *Tetranychus ludeni*. Means with the same letters are not significantly different (Tukey-Kramer test: *P* > 0.05). Female population density shift occurred on the 4th day of oviposition period

**Fig. 5:**
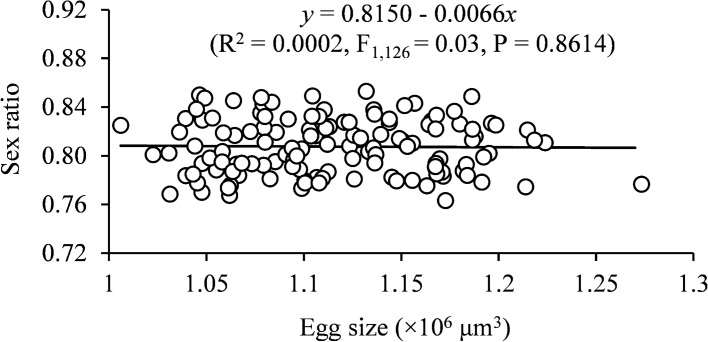
Relationships between sex ratio (proportion of female offspring) and egg size in *Tetranychus ludeni*

We demonstrate that the sex ratio (proportion of daughters) was significantly higher at high density than at low density regardless of shifts; density shift from high to high, from low to low, or from low to high significantly increased the sex ratio, and density shift from high to low significantly reduced the sex ratio (*F*_5,122_ = 11.26, *P* < 0.0001) (Fig. 4). Egg size had no significant impact on sex ratio (*F*_1,126_ = 0.03, *P* = 0.86) (Fig. 5).

## Discussion

Our results indicate that *T. ludeni* females reduced their fecundity after the population density changed during their productive period (Fig. 2a). We suggest that when the density quickly increases, they lower their fecundity to prevent the collapse of the local population due to the increase of resource competition and overexploitation of the host plants (Krips et al. 1998) or hostile interference or aggression among offspring for resource access (Estevez et al. 2007; Wong et al. 2013; Li and Zhang 2021). Tetranychid mites construct silk webs (Saito 1983; Mori and Saito 2005; Clotuche et al. 2009; Le Goff et al. 2010) in the new habitats to protect themselves and their offspring from environmental hazards (Davis 1952; McMurtry et al. 1970; Hazan et al. 1975; Ashley 2003; Oku et al. 2003, 2004; Mori and Saito 2005; Le Goff et al. 2010) but the silk consists of mainly proteins (Hazan et al. 1975), the production of which incurs a considerable cost (Oku et al. 2009). Therefore, when they arrive in a new site of much larger area from a higher density population, they need to allocate more resources per female to produce enough silk to cover the area, leading to fecundity decline (Fig. 2a).

We did not observe a trade-off between the egg number and size in response to the density changes (Fig. 3a), challenging theoretical assumptions (Smith and Fretwell 1974; Parker and Begon 1986; Roff 1992, 2002; Stearns 1992; Fox and Czesak 2000; Fischer et al. 2011). The lack of such trade-offs has also been reported in some animal species (e.g., Doughty and Shine 1997; Zera and Harshman 2001; Jordan and Snell 2002; Bowden et al. 2004; Uller and Olsson 2005). Our results show that increasing egg size did not significantly increase the proportion of daughters (Fig. 5), contradictory to the previous assumption that sex allocation in spider mites is mediated by egg size (Macke et al. 2011). These findings suggest that *T. ludeni* females only adjust their fecundity but not egg size in response to density dynamics as reported in some birds (Christians 2002) because egg size has little impact on reproductive fitness, such as offspring survival (Fig. 3b) and sex allocation (Fig. 5). Therefore, egg size is not a reliable indicator of offspring fitness when future environmental conditions are uncertain or unpredictable (Wiklund and Persson 1983; Karlsson and Wiklund 1985; McEdward and Carson 1987; Lalonde 2005; Morrongiello et al. 2012).

We demonstrate that regardless of density changes, offspring produced by females in high population density was significantly more female-biased than in low density (Fig. 4). This could be due to sex-specific dispersal tendency in spider mites. Female spider mites usually disperse from dense conditions after mating (Suski and Naegele 1968; McEnroe 1969; Brandenburg and Kennedy 1982; Li and Margolies 1993) to establish new colonies (Mitchell 1973; Brandenburg and Kennedy 1982) and reduce future competition for food or space (Clark 1978; Silk 1983; Mari et al. 2008; Hjernquist et al. 2009; West 2009; Visser et al. 2014; Song et al. 2016; Weerawansha et al. 2022), resulting in production of more dispersing daughters in dense conditions. Compared to density shift from high to high or from low to low, that from low to high led to a faster increase in proportion of daughters produced (Fig. 4). This suggests that *T. ludeni* females can quickly adjust their sex allocation in response to the change of social environment for optimal fitness of their offspring.

In the present study, we demonstrate that *T. ludeni* females could adjust their reproductive strategies in response to dynamic social environments during their reproductive period. Females reduce fecundity and produce more dispersive female offspring in dense environments, which will reduce the local resource competition. However, females do not adjust the egg size in response to the shift of population density, as egg size imposes no significant effect on fecundity and offspring sex ratio and survival. Therefore, *T. ludeni* females adapt to the shift of population density by manipulating the fecundity and offspring sex ratio but not the egg size. Whether *T. ludeni* females could manipulate sex allocation via adjusting egg size in response to the shift of population size remains unclear and is warranted for future investigations.
